# Genetic heterogeneity of cardiomyopathy and its correlation with patient care

**DOI:** 10.1186/s12920-023-01639-z

**Published:** 2023-10-30

**Authors:** Mi Jin Kim, Seulgi Cha, Jae Suk Baek, Jeong Jin Yu, Go Hun Seo, Minji Kang, Hyo-Sang Do, Sang Eun Lee, Beom Hee Lee

**Affiliations:** 1 Division of Pediatric Cardiology, Department of Pediatrics, Asan Medical Center, University of Ulsan College of Medicine, Seoul, Korea; 2 3billion, Inc, Seoul, Korea; 3 Genome Research Center for Birth Defects and Genetic Diseases, Asan Institute for Life Sciences, Asan Medical Center, Seoul, Korea; 4 Department of Cardiology, Asan Medical Center, University of Ulsan College of Medicine, Seoul, Korea; 5 Medical Genetics Center, Asan Medical Center Children’s Hospital, University of Ulsan College of Medicines, Seoul, Korea

**Keywords:** Cardiomyopathy, Whole-exome sequencing, Genotype, Prognosis

## Abstract

**Background:**

Cardiomyopathy, which is a genetically and phenotypically heterogeneous pathological condition, is associated with increased morbidity and mortality. Genetic diagnosis of cardiomyopathy enables accurate phenotypic classification and optimum patient management and counseling. This study investigated the genetic spectrum of cardiomyopathy and its correlation with the clinical course of the disease.

**Methods:**

The samples of 72 Korean patients with cardiomyopathy (43 males and 29 females) were subjected to whole-exome sequencing (WES). The familial information and clinical characteristics of the patients were reviewed and analyzed according to their genotypes.

**Results:**

Dilated cardiomyopathy (DCM), hypertrophic cardiomyopathy (HCM), left ventricular non-compaction cardiomyopathy, and restrictive cardiomyopathy was detected in 41 (56.9%), 25 (34.7%), 4 (5.6%), and 2 (2.8%) patients, respectively. WES analysis revealed positive results in 37 (51.4%) patients. Subsequent familial testing identified ten additional familial cases. Among DCM cases, 19 (46.3%) patients exhibited positive results, with *TTN* variants being the most common alteration, followed by *LMNA* and *MYH7* variants. Meanwhile, among HCM cases, 15 (60%) patients exhibited positive results with *MYH7* variants being the most common alteration. In six patients with positive results, extracardiac surveillance was warranted based on disease information. The incidence of worse outcomes, such as mortality and life-threatening arrhythmic events, in patients with DCM harboring *LMNA* variants, was higher than that in patients with DCM harboring *TTN* or *MYH7* variants.

**Conclusions:**

Diverse genotypes were identified in a substantial proportion of patients with cardiomyopathy. Genetic diagnosis enables personalized disease surveillance and management.

**Supplementary Information:**

The online version contains supplementary material available at 10.1186/s12920-023-01639-z.

## Background

Cardiomyopathy is a pathological condition characterized by progressive cardiac muscle weakness. The prevalence of cardiomyopathy, which varies according to the sub-phenotype, in the general population is estimated to be approximately 0.004–0.2% [1]. Familial cardiomyopathy accounts for 20–50% of all cardiomyopathy cases, suggesting an underlying genetic predisposition [2–4].

More than 60 genes have been reported to be associated with cardiomyopathy [5]. Genetic diagnosis enables physicians to accurately delineate the phenotypic classification of cardiomyopathy in affected patients and provide personalized disease surveillance and management. Moreover, genetic counseling for the family members at risk will aid in identifying cardiomyopathy in asymptomatic or presymptomatic subjects and consequently enable early intervention to delay disease progression or reduce the risk of sudden cardiac death [6]. Based on the wide range of penetrance and expressivity of inherited cardiomyopathy, early identification of pre-symptomatic patients is critical for improving disease prognosis [7]. Therefore, current medical guidelines recommend genetic testing with cardiac surveillance for patients with suspected cardiomyopathy and their first-degree relatives.

The development of next-generation sequencing technology has enabled the genetic diagnosis of cardiomyopathy [8]. Testing a specific panel of genes has usually been recommended for well-defined phenotypes with locus heterogeneity. Recently, whole-exome sequencing (WES) and whole-genome sequencing have been widely used for molecular diagnosis [9, 10].

In this study, the samples of 72 Korean patients with cardiomyopathy were subjected to WES. Additionally, the genetic spectrum of cardiomyopathy and its clinical applications have been discussed.

## Methods

### Patient selection and genetic analysis

The study population comprised 72 patients with cardiomyopathy (both familial and non-familial cases) who were prospectively enrolled at the Asan Medical Center, Seoul, Korea, between January 2018 and December 2021. The enrolled patients did not exhibit (i) congenital heart disease, (ii) primary valvular disease, (iii) drug- or infection-related myocarditis, (iv) myocardial infarction with significant coronary artery disease, or postpartum cardiomyopathy [11]. Cardiomyopathy was defined and classified based on the guidelines of the European Society of Cardiology as follows: dilated cardiomyopathy (DCM), hypertrophic cardiomyopathy (HCM), left ventricular non-compaction (LVNC) cardiomyopathy, or restrictive cardiomyopathy (RCM) [12].

Clinical findings, such as demographic information, family history with pedigree; whether there were family members with (1) diagnosed cardiomyopathy, (2) clinical recommendations including drugs or devices, and (3) sudden cardiac death before the age of 50 years, cardiomyopathy class, the results of cardiac surveillance (echocardiograms, electrocardiograms, Holter monitoring, cardiac magnetic resonance imaging, and endomyocardial biopsies), hospitalizations, cardiac transplant status, and cause of death, were reviewed based on the medical record. Informed consent was obtained from all the participants and the parents of the patients for the genetic test. The Institutional Review Board of the Human Research of Asan Medical Center(IRB numbers: 2018 − 0574 and 2018 − 0180) and the Asan Institute for Life Sciences (Seoul, Republic of Korea) (20211P003) approved this study. This study was performed according to the Declaration of Helsinki and approved by the institutional Medical Ethics Committee.

### Genetic analysis

Genomic DNA isolated from the whole blood samples or buccal swab samples of patients was subjected to WES. All exons of all human genes were captured using a Twist Human Core Exome kit (Twist Bioscience, San Francisco, CA, USA). The genomic regions were sequenced using a NovaSeq 6000 platform (Illumina, San Diego, CA, USA). Raw genome sequencing data analysis included alignment to the reference sequence (National Center for Biotechnological Information genome assembly GRCh37; accessed in February 2009). The mean depth of coverage was 100-fold, with 99.2% higher coverage than 10-fold. All detected variants were confirmed using Sanger sequencing. Relevant patient phenotypes were assessed using the automated variant interpretation system EVIDENCE [13]. Genetic variants were classified into the following five classes according to the criteria proposed by the American College of Medical Genetics and Genomics (ACMG) guidelines: pathogenic, likely pathogenic, variants of unknown significance (VUS), likely benign, or benign. Both pathogenic and likely pathogenic variants were considered pathogenic variants, while all other variants were considered non-pathogenic variants [14].

### Statistical analysis

Descriptive statistics are represented as counts and percentages for categorical variables and mean ± standard deviation or median with interquartile range (IQR) for continuous variables. Intergroup comparison was performed using the Chi-square test and Fisher’s exact test for categorical variables and the Student’s t-test or Wilcoxon rank sum test for continuous variables. Clinical outcomes of patients with DCM were compared using the Kruskal-Wallis test, followed by Dunn’s posthoc test according to the genotype. Differences were considered significant at p < 0.05. All statistical analyses were performed using SPSS 21.0 (IBM Corp., Armonk, NY, USA) or R software (version 3.6.3; www.r-project.org).

## Results

### Clinical characteristics of patients with cardiomyopathy

This study included 72 patients (43 males (59.7%) and 29 females (40.3%)) (Table 1). Of these, 35 patients (48.6%) were familial cases. The median ages at diagnosis and enrolment were 29 (IQR = 12–41) and 34.5 (IQR = 15.8–45) years, respectively. DCM and HCM were diagnosed in 41 (56.9%) and 25 (34.7%) patients, respectively. LVNC cardiomyopathy and RCM were diagnosed in 4 (5.6%) and 2 (2.7%) patients, respectively. (Supplemental Fig. 1) The baseline clinical and echocardiography findings are summarized in Table 1. The age at diagnosis in patients with HCM (n = 25; 16 years (IQR = 0–45.8)) was lower than that in patients with DCM (n = 41; 30.1 years (16–39)) (p = 0.008). The number of familial cases among patients with DCM (26/41 patients; 63.4%) was higher than that among patients with HCM (8/25 patients; 32%) (p = 0.013). Compared with those in patients with HCM, the left ventricular ejection fraction (30.73% ± 20.08% vs. 60.07% ± 23.01%; p = 0.001) was lower and the left ventricular end-diastolic diameter was higher (54.84 mm ± 16.25 mm vs. 38.82 mm ± 16.41 mm, p = 0.001). In contrast, the interventricular septum thickness (14.91 mm ± 7.76 mm vs. 7.46 mm ± 2.0 mm, p = 0.001) and left ventricular mass index (186.05 g/m^2^ ± 93.94 g/m^2^ vs. 108.15 g/m^2^ ± 29.24 g/m^2^, p = 0.039) in patients with HCM were higher than those in patients with DCM. The number of males and females (p = 0.36) and the proportion of patients with limiting daily activities of New York Heart Association (NYHA) class III or higher (p = 0.851) and the incidence of family history of sudden death at < 50 years of age (p = 0.473) were not significantly different.

**Table 1:** Clinical and echocardiographic findings of patients with cardiomyopathy

	All (n = 72; DCM, HCM, RCM, LVNC)	Type of cardiomyopathy			
		DCM (n = 41; 56.9%)	HCM (n = 25; 34.7%)	RCM, (n = 2; 2.8%)	LVNC (n = 4; 5.6%)
Male, n (%)	43 (59.7)	26 (63.4)	13 (52)	2 (100)	2 (50)
Familial, n (%)	35 (48.6)	26 (63.4)	8 (32)	0 (0)	1 (25)
Familial history of sudden death, n (%)	21 (29.2)	15 (36.6)	6 (24)	0 (0)	0 (0)
NYHA ≥ 3, n (%)	15 (20.8)	9 (21.9)	5 (20)	1 (50)	0 (0)
Median (IQR) age at diagnosis, years	29 (12–41)	**30.1 (16–39)**	**16 (0–45.8)**	60 (52–68)	7.6 (0–18.3)
Median (IQR) age at enrolment, years	34.5 (15.8–45)	**36 (28–41)**	**20 (3.6–54)**	61 (53–69)	13 (5.4–21.5)
Median (IQR) time from diagnosis to enrolment, years	3 (1–5)	3 (1–5)	2.9 (1.4–5)	1 (1–1)	2.45 (0.9–4.7)
Echocardiography					
LVEF (%)	41.8 ± 21.26	**30.73 ± 10.08**	**60.07 ± 23.01**	39 ± 18.39	36.5 ± 19.13
LVEDD (mm)	48.4 ± 17.78	**54.84 ± 16.25**	**38.82 ± 16.41**	16.41 ± 48	44.8 ± 23.46
IVSd (mm)	10.16 ± 5.98	**7.46 ± 2.0**	**14.91 ± 7.76**	11 ± 1.41	7.13 ± 3.0
PWDd (mm)	8.03 ± 3.17	6.1 ± 3.27	8.43 ± 4.53	12.5 ± 0.71	6.6 ± 2.6
LV mass index (g/m²)	135.68 ± 124.09	**108.15 ± 29.24**	**186.05 ± 93.94**	111.5 ± 18.38	85.31 ± 24.97
Restrictive mitral pattern (%)	18 (25)	10 (24.4)	7 (28)	1 (50)	0 (0)
Mitral valve regurgitation ≥ moderate	21 (29.2)	15 (36.6)	6 (24)	1 (50)	0 (0)
Follow-up data					
Anti-arrhythmic agent	9 (12.5)	4 (9.8)	5 (20)	0 (0)	0 (0)
P-M or ICD or CRT implantation	7 (9.7)	4 (9.8)	3 (12)	0 (0)	0 (0)
Any device (VAD or ECMO)	5 (6.9)	4 (9.8)	1 (4)	0 (0)	0 (0)
Heart transplantation	10 (13.9)	**9 (22)**	**0 (0)**	1 (50)	0 (0)
Mortality	8 (11.1)	4 (9.8)	3 (12)	0 (0)	1 (25)
Mean follow-up duration (years)	4.5	4.97	4.21	1	3.15

DCM, dilated cardiomyopathy; HCM, hypertrophic cardiomyopathy; RCM, restrictive cardiomyopathy; LVNC, left ventricular non-compaction; NYHA, New York Heart Association; IQR, interquartile range; LV, left ventricular; EF, ejection fraction; EDD, end-diastolic diameter; IVSd, interventricular septum diameter at diastole; PWDd, posterior wall diameter at diastole; P-M, pacemaker; ICD, implantable cardioverter defibrillator; CRT, cardiac resynchronization therapy; VAD, ventricular assist device; ECMO, extracorporeal membrane oxygenation. Bold characters represent p < 0.05 (patients with HCM vs. patients with DCM)

During the follow-up period of 4.5 years (IQR = 1–5 years), various arrhythmic events developed in 9 (12.5%) patients. Among them, 7 (9.7%) patients received implantation of cardiac assistance devices, such as a pacemaker (5 patients; 6.9%), an implantable cardioverter defibrillator (ICD) (1 patient; 1.4%), or cardiac resynchronization therapy (CRT) (1 patient; 1.4%) for life-threatening arrhythmic events such as non-fatal ventricular fibrillation and/or sustained ventricular tachycardia. Heart failure progressed into NYHA Class IV in 5 patients who were implanted with a ventricular assist device (VAD) or supported with extracorporeal membrane oxygenation before heart transplantation or death. Of the 10 (13.9%) patients who underwent heart transplantation, 9/41 (22%) and 1/2 (50%) patients were DCM and RCM cases. However, none of the patients with HCM underwent heart transplantation (0/25 patients; 0%) (p = 0.012). Among the patients who underwent heart transplantation, 8 survived for an average of 2 years (IQR = 0–4 years) after the transplantation.

### Genetic diagnosis of patients with cardiomyopathy

**Fig. 1:**
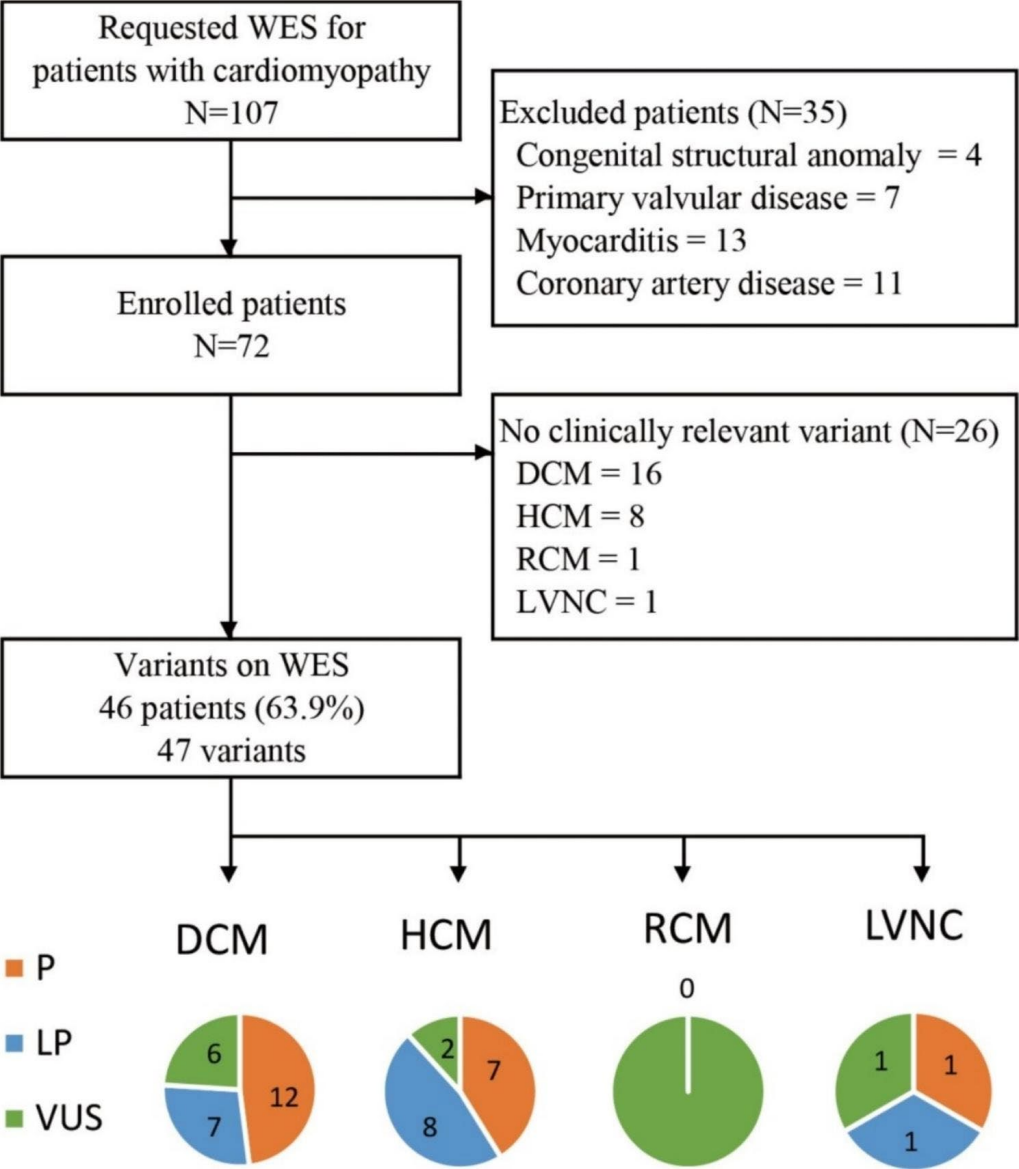
The process of diagnosing patients with cardiomyopathy. Abbreviations: WES, whole-exome sequencing; P, pathogenic; LP, likely pathogenic; VUS, variant of unknown significance; DCM, dilated cardiomyopathy; HCM, hypertrophic cardiomyopathy; RCM, restrictive cardiomyopathy; LVNC, Left ventricular non-compaction

The samples of all 72 patients were subjected to WES. The common variants with a minor allele frequency of > 5% were filtered out. Only the variants in the genes that matched the phenotype of the patients were selected. Among them, likely benign, benign, and non-coding variants with low evidence were excluded according to the ACMG guidelines [15]. Only pathogenic or likely pathogenic variants were reported as positive results. These variants were detected in 37 (51.4%) patients (pathogenic variants in 20/72 patients (27.8%) and likely pathogenic variants in 17/72 patients (23.6%)). VUS were detected in 9/72 patients (12.5%) (Supplemental Table 1). Among the 41 patients with DCM, 19 (46.3%) exhibited positive results (pathogenic variants in 12 patients (29.3%) and likely pathogenic variants in 7 patients (17.1%)), while 6 (14.6%) patients harbored VUS. Meanwhile, among the 25 patients with HCM, 15 patients (60%) exhibited positive results (pathogenic variants in 7 patients (28%) and likely pathogenic variants in 8 patients (32%)), while 2 (8%) patients harbored VUS (Fig. 1).

In familial cases, 7/26 (26.9%) and 3/8 (37.5%) patients with DCM and HCM exhibited positive results, respectively.

The diagnostic yield was different according to the severity of the disease, which was 73.3% in the 15 patients with limiting daily activities of New York Heart Association (NYHA) class III or higher at the time of study participation. On the other hand, among 57 patients with symptoms below NYHA class III, 26 patients (45.6%) had pathogenic or likely pathogenic variants.

The frequency of extracardiac or syndromic clinical manifestations, including other major malformations, intellectual disability or autism, global developmental delay, or growth deficits in patients with HCM (6/15 patients; 40%) was higher than that in patients with DCM (0/19 patients; 0%) (p = 0.009). Five patients were diagnosed with rare syndromic disorders; Danon disease (1 patient), Costello syndrome (1 patient), and Noonan syndrome (3 patients) (Table 2).

**Table 2:** Extracardiac manifestations in patients with cardiomyopathy exhibiting syndromic disorders

No	Gene	Cardiac manifestations	Extracardiac manifestations
1	*LAMP2*	HCM	Danon disease, peripheral pigmentary retinopathy, and proximal muscle weakness
2	*PTPN11*	HCM	Noonan syndrome, SNHL, Café au lait sports, hypertelorism, and flat nasal root
3	*HRAS*	HCM	Costello syndrome, macrocephaly, spare hair and eyebrow, short neck, epicanthal fold, proptosis, clenched hand, low set ear, micrognathia, widely spaced nipple, and cystic hygroma
4	*MYH7*	HCM	Urothelial carcinoma
5	*BRAF*	HCM	Noonan syndrome, CFC, hypotonia fragile hair, low set ear, and webbed neck
6	*PTPN11*	HCM	Noonan syndrome, ACC, exotropia, SDH, SAH, both renal mild hydronephrosis, large ASD, cryptorchidism, thrombocytopenia, hydrops

HCM, hypertrophic cardiomyopathy; SNHL, sensorineural hearing loss; CFC, cardiofaciocutaneous; ACC, agenesis of corpus callosum; SDH, subdural hemorrhage; SAH, subarachnoid hemorrhage; ASD, atrial septal defect

The following 13 variants were not previously reported: two TTN variants (c.83994del and c.6621del), three MYH7 variants (c.1182 C > A, c.1548 C > A, and c.602T > C), and one TNNT2 (c.517_519del), one VHL (c.458_470dup), one KCNE1 (c.386dup), one TNNI3 (c.512 C > A) one DSP (c.5126_5127del), one MYLK2 (c.1577 + 1G > A), one LAMP2 (c.123 C > A), and one MYBPC3 (c.2067 + 1G > A) variants (Supplemental Table 2).

### Genotype-phenotype correlations

**Fig. 2:**
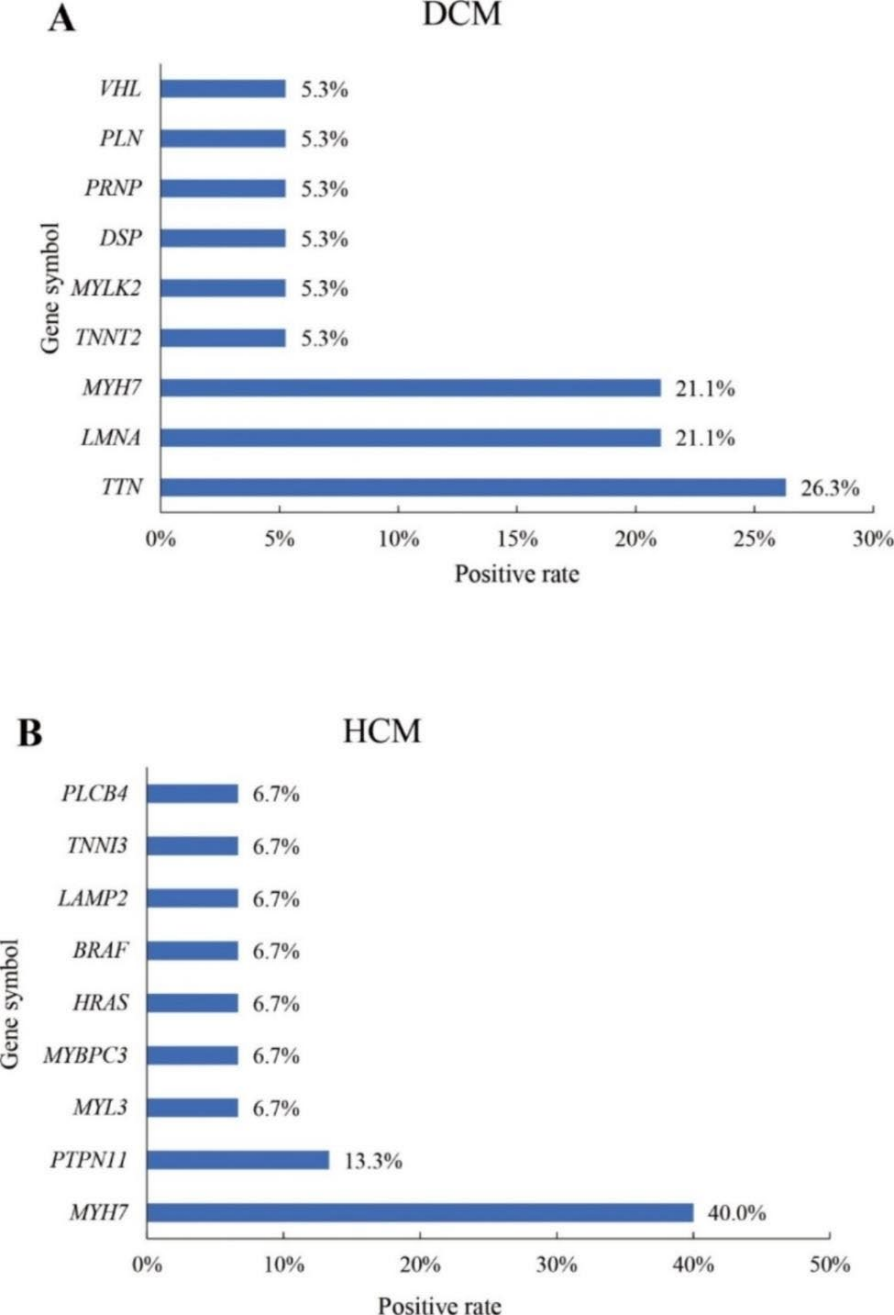
Genotype spectra of dilated cardiomyopathy (DCM) (**A**) and hypertrophic cardiomyopathy (HCM) (**B**)

Among 19 patients with DCM exhibiting positive results, TTN variants (5/19 pts, 26.3%) were most frequently detected, followed by LMNA (4/19 patients; 21.1%), MYH7 (4/19 patients; 21.1%) variants and TNNT2 (1/19 patients; 5.3%), DSP (1/19 patients; 5.3%), PRNP (1/19 patients; 5.3%), PLN (1/19 patients; 5.3%), VHL (1/19 patients; 5.3%)and MYLK2 (1/19 patients; 5.3%) variants (Fig. 2). Meanwhile, among the 15 patients with HCM exhibiting positive results, MYH7 (6/15 patients; 40%) variant was most frequently detected. PTPN11 (2/15 patients; 13.3%), MYL3 (1/15 patients; 6.7%), MYBPC3 (1/15 patients; 6.7%), HRAS (1/15 patients; 6.7%), BRAF (1/15 patients; 6.7%), TNNI3 (1/15 patients; 6.7%), PLCB4 (1/15 patients; 6.7%) and LAMP2 (1/15 patients; 6.7%) variants were also identified in patients with HCM.

The clinical findings were compared among the patients with three common genotypes (TTN, LMNA, and MYH7 variants). Familial history, sudden cardiac death history, NYHA function class at the time of diagnosis, and the hemodynamical state (based on echocardiography results obtained at the time of diagnosis) were not significantly different (Table 3).

**Table 3:** Clinical outcomes of patients with dilated cardiomyopathy (DCM) caused due to *TTN*, *LMNA*, or other gene variants

	Total (n = 15)	*TTN* (n = 5)	*LMNA* (n = 4)	*MYH7* (n = 4)
Age at diagnosis (years)	31.01 ± 15.92	29.82 ± 10.28	33.75 ± 9.39	**26.75 ± 18.45**
Male, n (%)	11 (73.3)	3 (50)	4 (100)	**3 (75)**
Familial, n (%)	11 (73.3)	3 (60)	4 (100)	**3 (75)**
Familial history of sudden death, n (%)	7 (46.7)	3 (60)	3 (75)	**1 (25)**
NYHA function class ≥ 3, n (%)	2 (13.3)	1 (20)	1 (25)	**0 (0)**
Echocardiography findings				
LVEF (%)	34.82 ± 13.83	34.02 ± 11.48	45.2 ± 11.4	**26.15 ± 6.17**
LVEDD (mm)	59.22 ± 28.48	61.24 ± 5.11	58.73 ± 4.94	**50.03 ± 19.92**
LVESD (mm)	47.35 ± 9.68	49.32 ± 6.74	43.43 ± 5.98	**42.8 ± 16.78**
LV mass index (g/m²)	107.79 ± 49.1	108.28 ± 20.79	97.02 ± 20.16	**101.72 ± 8.92**
Restrictive mitral pattern (%)	4 (26.7)	1 (20)	0 (0)	**2 (50)**
Mitral valve regurgitation ≥ moderate	4 (26.7)	2 (40)	1 (25)	**0 (0)**
Follow-up data				
Anti-arrhythmic agent, n (%)	3 (20)	**0 (0)**	**3 (75)**	**0 (0)**
P-M or ICD or CRT implantation, n (%)	4 (26.7)	1 (20)	**2 (50)**	**0 (0)**
Any device (VAD or ECMO), n (%)	4 (26.7)	1 (20)	3 (75)	**0 (0)**
Heart transplantation, n (%)	4 (26.7)	2 (40)	1 (25)	**0 (0)**
Mortality, n (%)	2 (13.3)	**0 (0)**	**2 (50)**	**0 (0)**

DCM, Dilated cardiomyopathy; LV, left ventricular; EF, ejection fraction; EDD, end-diastolic diameter; ESD, end-systolic diameter; P-M, pacemaker; ICD, implantable cardioverter defibrillator; CRT, cardiac resynchronization therapy; VAD, ventricular assist device; ECMO, extracorporeal membrane oxygenation. Bold characters represent p < 0.05 for comparison between genotypes

**Fig. 3:**
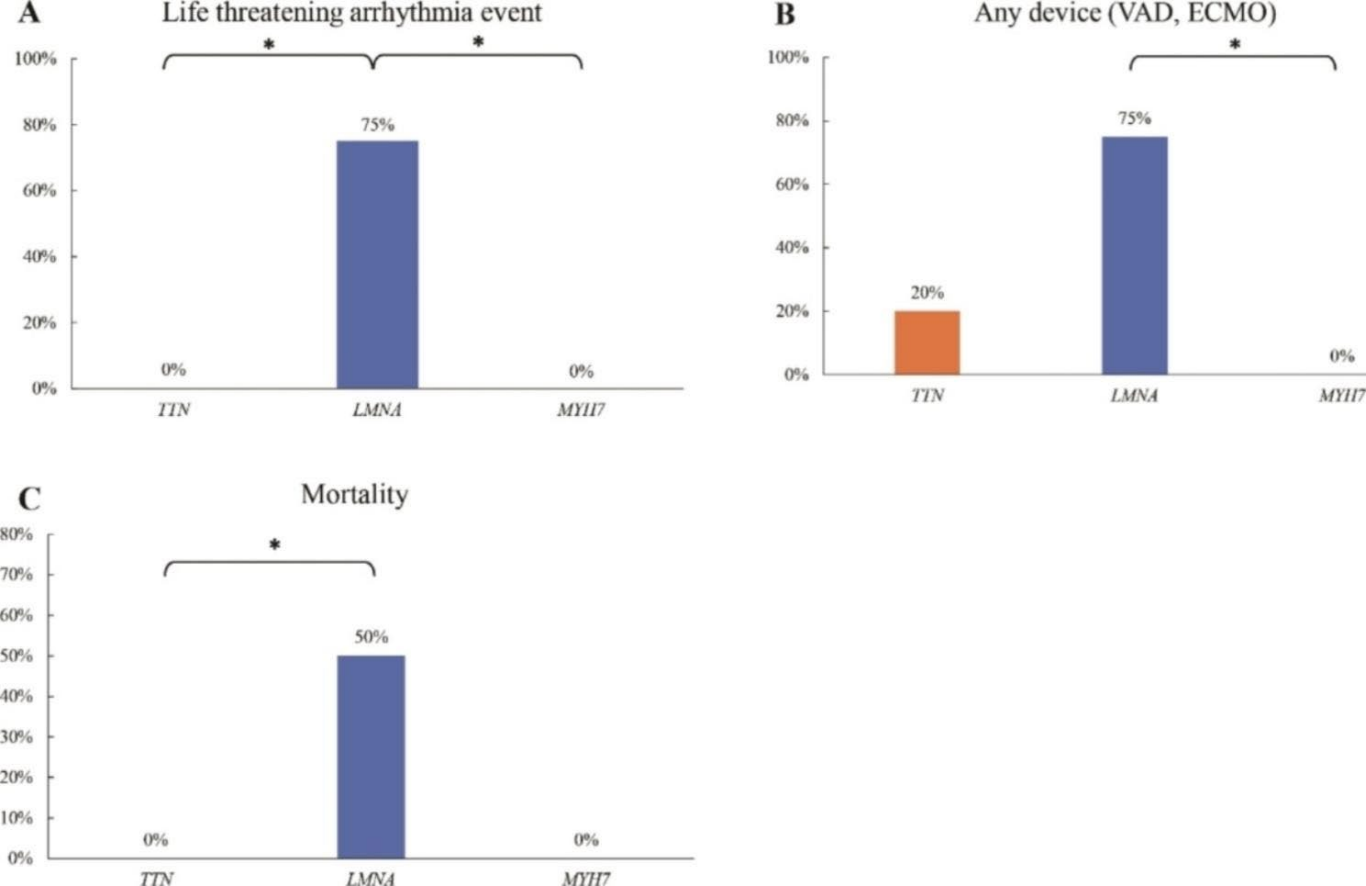
Clinical outcomes of patients with dilated cardiomyopathy (DCM) harboring *TTN*, *LMNA*, and *MYH7* variants. **A**. The incidence of life-threatening arrhythmia events was significantly higher in patients with DCM harboring *LMNA* variants; **B**. The need for VAD insertion or ECMO support in patients with DCM harboring *LMNA* variants was higher than that in patients with DCM harboring *MYH7* variants; **C**. All patients who died during the follow-up period were DCM cases harboring *LMNA* variants, and the mortality rate in these patients was significantly higher than that in patients harboring *TTN* variants. *p < 0.05 (Fisher’s exact test). Abbreviations: DCM, dilated cardiomyopathy; VAD, ventricular assist device; ECMO, extracorporeal membrane oxygenation

In patients with DCM, the incidence of a life-threatening arrhythmia (3/4 patients; 75%) in patients harboring LMNA variants was higher than that in patients harboring TTN (0/5 patients; 0%) (p = 0.008) or MYH7 (0/4 patients; 0%; p = 0.02) variants (Fig. 3). Additionally, the percentage of patients who were implanted with VAD among DCM cases (3/4 patients; 75%) harboring LMNA variants was higher than that among DCM cases with MYH7 variants (0/4 patients; 0%) (p = 0.02). Additionally, the death rate in patients with DCM harboring LMNA variants (2/4 patients; 50%) was higher than that in patients with DCM harboring TTN variants (0/5 patients; 0%) (p = 0.046).

### Clinical application of genetic testing

For patients who had positive results, testing of family members was recommended. Among the 35 patients with familial cardiomyopathy, the families of 32 patients were screened. Of these 32 patients, 4 were confirmed to have genetic variants but did not exhibit symptoms of cardiomyopathy at the time of genetic confirmation. Two patients (6.25%) had pathogenic variants, while two patients (6.25%) had likely pathogenic variants. The patients had no limitations in their daily activities and did not exhibit deterioration in exercise ability to the extent that they could be classified as NYHA Class I. However, echocardiography revealed left ventricular dilatation and borderline systolic dysfunction in 2 patients. Following the diagnosis, patients were advised to make some lifestyle changes, particularly to avoid competitive sports, and instead opt for aerobic activities such as walking (outside or on a treadmill), stationary cycling, swimming, rowing, or aqua aerobics. In two patients with systolic dysfunction, cardiac surveillance medical therapy was initiated for primary prevention and to prevent the syndrome of clinical heart failure, according to American Heart Association guidelines [16]. Periodic echocardiography and electrocardiography were scheduled to monitor cardiac function.

Extracardiac surveillance was performed for 6 patients with syndromic disorders. Ophthalmological examination revealed pigmented retinopathy in a 23-year-old female patient diagnosed with Danon disease [17]. In three patients with Noonan syndrome, extracardiac anomalies were examined, and growth and developmental status were monitored regularly. One patient was diagnosed with Costello syndrome, exhibiting characteristic facial musculoskeletal aberrations, cystic hygroma, and macrocephaly. Another patient was diagnosed with urothelial carcinoma via extracardiac surveillance.

One DCM case and one LVNC cardiomyopathy case with positive results were consulted for pregnancy. The eligibility for prenatal or preimplantation genetic testing was discussed from the perspective of South Korean laws. Additionally, the risk-to-benefit ratio of the use of anti-arrhythmic and anti-heart failure s during pregnancy was discussed.

## Discussion

Various factors, such as genetic, epigenetic, and environmental factors, determine the phenotype of cardiomyopathy. The elucidation of genetic factors will improve our understanding of the phenotypic differences in cardiomyopathies and determine the treatment direction and prognosis [18]. This study investigated the genetic spectrum of cardiomyopathy in Korean patients and its applicability in clinical care.

Consistent with the findings of the previous studies, DCM (41/72 patients; 56.9%) and HCM (25/72 patients; 34.7%) were the most common subtypes, whereas RCM (2/72 patients; 2.8%) and isolated LVNC cardiomyopathy (4/72 patients; 5.6%) were rare subtypes [19, 20]. The genetic diagnosis rates of DCM, HCM, LVNC cardiomyopathy, and RCM in this study were 46.3% (19/41 patients), 60% (15/25 patients), 50% (2/4 patients), and 50% (1/2 patients), respectively. The positive result rate for DCM obtained in this study was similar to that obtained by Pugh et al. [21] (37%) but was lower than that obtained by other studies (57–73%) [22, 23]. Additionally, the positive result rate for HCM obtained in this study was slightly higher than that obtained in previous studies (22–34%). The diagnostic rate of LVNC cardiomyopathy has been reported to be 30–50%, whereas that of RCM has not been determined. These diverse genetic diagnostic results among the subtypes can be attributed to the difference in the race/ethnicity of the study cohort, the number of genes tested in each study, or the modality of the next-generation sequencing techniques applied [24].

In this study, the age of the subjects at diagnosis was 29 years (IQR = 12–41 years), while that at the time of the request for genetic testing was 34.5 years (IQR = 15.8–45 years). Although the time of diagnosis varies depending on race and phenotype, previous studies have reported that the time of onset of symptoms due to cardiomyopathy in adults is in the range of 20–60 years [25]. The timing of when genetic testing is indicated for patients with cardiomyopathy has not been studied. However, genetic testing is commonly recommended for diagnosis owing to its rapid expansion [26].

In addition, higher diagnostic yield was detected in our patients with NYHA class III/IV as in previous studies [13, 27], warranting the genetic evaluation in the patients with severe cardiac dysfunction.

The genetic spectrum of cardiomyopathies varies according to the subtype [8, 15, 21, 28]. In DCM, genetic variants have been most commonly identified in TTN (15–25%) [28, 29] and LMNA (5–10%), followed by sarcomere-related genes (such as MYH6, MYH7, MYBPC3, TNNT2, TNNI3, TNNC1, TPM1, and ACTC1) (10%) [30] and desmosome-related genes (DSP and PKP2) (5%) [31]. This study demonstrated that the mutation spectrum was similar in TTN (26.3%), LMNA (21.1%), MYH7 (21.1%), TNNT2 (5.3%), DSP (5.3%), MYLK2 (5.3%), PRNP (5.3%), PLN (5.3%), and VHL (5.3%). In HCM, genetic variants have been most commonly identified in MYBPC3 (30–40%) and MYH7 (20–30%), followed by TNNT2 (10%), TNNI3 (7%), MYL2, MYL3, TPM1, and ACTC1 [8, 32, 33]. Although MYH7 variants (40%) were the most common alterations detected in this study, PTPN11 (13.3%), HRAS (6.7%), BRAF (6.7%), MYL3 (6.7%), MYBPC3 (6.7%), TNNI3 (6.7%), PLCB4 (6.7%),and LAMP2 (6.7%) variants, which have been reported as rare variants, were also detected [32–34]. This difference may be related to the phenotypes of the patients, as these variants are associated with syndromic disorders such as Noonan, Costello, cardiofaciocutaneous, or Danon syndrome.

The results of genetic testing provide important information for the management of patients. For example, patients with DCM harboring the LMNA or PLN variants have a high risk for end-stage heart failure and life-threatening arrhythmia [22, 35], whereas those harboring the TTN variants exhibit good responses to medical treatment such as beta-blockers, antiotensin-coverting enzyme inhibitors, angiotensin II receptor blockers (ARBs), diuretics and ivabradine [28, 36]. Similar findings were observed among the patients enrolled in this study. Therefore, an ICD implantation and or early registration for heart transplantation should be considered for patients with DCM harboring the LMNA variant, especially for those with serious risk factors, such as non-sustained ventricular tachycardia, a left ventricular ejection fraction below 45% at first evaluation, male sex, and non-missense mutations. Patients with HCM harboring MYH7 variants exhibited severe ventricular hypertrophy and ventricular exacerbation when compared with those harboring other genetic variants. Hence, careful monitoring of conditions such as life-threatening arrhythmia and hypertension is required for these patients.

The variants of the same gene do not cause the same degree of cardiac dysfunction. In fact, among our patients, different phenotypes were observed according to the different genotypes of the MYH7 gene; DCM (24% of LVEF) due to the c.1357 C > T (p.Arg453Cys) variant and HCMP (68% of LVEF) due to the c.1988G > A (p.Arg663His) variant. It is worth to investigate this phenotypic heterogeneity among the diverse genotypes in a single gene.

In some cases, life-threatening arrhythmias or sudden cardiac death could be the first symptoms of cardiomyopathy, and genetic testing of family members might be overlooked in clinical practice. Hence, family history information should be obtained and the pedigree of family members spanning at least three generations should be investigated. In addition, appropriate genetic testing for at-risk family members should be performed [37]. In this study, four pathogenic variants were identified in a family screening test of 32 patients with cardiomyopathy.

The results of this study indicate that extracardiac surveillance is required for some patients with variants involved in the pathogenesis of syndromic disorders, such as Noonan, Costello, cardiofaciocutaneous, or Danon syndrome [19]. Other rare syndromic genetic defects, such as GLA (0.4–1%) [34, 38], TTR (0.6%) [39], and PRKAG2 (0.4%) [34], have been previously reported but were not reported in this study. Surveillance and appropriate management of extracardiac manifestations are important for these patients with syndromic disorders.

Clinical application of genetic testing will provide useful information to family members on reproductive options through preimplantation or prenatal genetic screening, as well as postpartum genetic testing and an opportunity to consider the benefits and potential harms of each option.

This study identified 13 previously unreported disease-causing variants (4 pathogenic variants and 9 likely pathogenic variants) in 13 patients. In total, 9 VUS were identified in the study cohort (9/72 patients; 12.5%). As the pathogenic role of VUS is unknown due to insufficient evidence, future studies must evaluate its role in the pathogenesis of cardiomyopathy.

This study has several limitations. In this study, a small number of patients were recruited from a single center. Additionally, the samples from the enrolled patients were evaluated using only WES. Thus, the overall diagnosis rate and genetic spectrum may vary when a large cohort is analyzed. Furthermore, although this study approached all eligible patients with cardiomyopathy, the possibility of survivor bias and a potential selection bias cannot be ruled out.

In conclusion, advances in genetic testing have enabled the identification of genetic factors in cardiomyopathy, which will improve the clinical application of genetic diagnosis.

### Electronic supplementary material

Below is the link to the electronic supplementary material.

Supplementary Material 1

Supplementary Material 2

Supplementary Material 3

## Data Availability

Reference sequences for *ARID1B* (NC_000006.12), *SMARCA4* (NC_000019.10), *SMARCB1* (NC_000022.11), *SMARCA2* (NC_000009.12), and *ARID2* (NC_000012.12) are available in the GenBank repository. The links to the GenBank repositories are as follows; *ARID1B* (https://www.ncbi.nlm.nih.gov/nuccore/NC_000006.12?from=156776026&to=157210779&report=genbank), *SMARCA4* (https://www.ncbi.nlm.nih.gov/nuccore/NC_000019.10?from=10960999&to=11062277&report=genbank), *SMARCB1* (https://www.ncbi.nlm.nih.gov/nuccore/NC_000022.11?from=23786966&to=23838009&report=genbank), *SMARCA2* (https://www.ncbi.nlm.nih.gov/nuccore/NC_000009.12?from=2015347&to=2193624&report=genbank), *ARID2* (https://www.ncbi.nlm.nih.gov/nuccore/NC_000012.12?from=45729706&to=45908037&report=genbank). Databases used in this study were Human Gene Mutation Database (HGMD, http://www.hgmd.cf.ac.uk), ClinVar database (https://www.ncbi.nlm.nih.gov/clinvar), gnomAD Browser (https://gnomad.broadinstitute.org/), SIFT (http://provean.jcvi.org/index.php), PROVEAN (http://provean.jcvi.org/index.php), PolyPhen-2 (http://genetics.bwh.harvard.edu/pph2/), and MutationTaster (http://www.mutationtaster.org/).

## Abbreviations

WES: Whole-exome sequencing
DCM: Dilated cardiomyopathy
HCM: Hypertrophic cardiomyopathy
RCM: Restrictive cardiomyopathy
LVNC: Left ventricular non-compaction
VUS: Variants of unknown significance
IQR: Interquartile range
