# Supplementary material for: Genetic heterogeneity of cardiomyopathy and its correlation with patient care
Source: BMC Med Genomics. 2023 Oct 30;16:270. doi: 10.1186/s12920-023-01639-z (PMC10614404; doi:10.1186/s12920-023-01639-z)
Supplement: Supplementary file 1 — Supplementary Material 1 [file 12920_2023_1639_MOESM1_ESM.docx]

Supplemental Table 2. Novel genetic variations detected in 13 patients with cardiomyopathy.

| Patient No. | Gene name | Position | Nucleotide change | Protein change | GenBank number | Predicted pathogenicity† | Genetic origin | ACMG |
| --- | --- | --- | --- | --- | --- | --- | --- | --- |
| 1 | *TNNT2* | chr1:201332504 | c.517_519del | p.Glu173del | NM_001276345.1 | Pathogenic | _‡ | PS4_S, PP1_S, PM2_M, PM4_ |
| 2 | *TTN* | chr2:179426864 | c.83994del | p.Asp27999MetfsTer19 | NM_001267550.2 | Likely pathogenic | Paternal | PVS1_S, PM2_M |
| 3 | *TTN* | chr2:179639816 | c.6621del | p.Trp2207CysfsTer28 | NM_001267550.2 | Likely pathogenic | _ | PVS1_S, PM2_M |
| 4 | *DSP* | chr6:7581544 | c.5126_5127del | p.Leu1709HisfsTer24 | NM_004415.4 | Likely pathogenic | _ | PVS1_VS, PM2_M |
| 5 | *MYLK2* | chr20:30419659 | c.1577+1G>A |  | ENST00000375994.2 | Likely pathogenic | Maternal | PM2_M |
| 6 | *MYH7* | chr14:23898513 | c.1182C>A | p.Asp394Glu | NM_000257.3 | Pathogenic | Paternal | PS4_S, PM1_M, PM2_M, PM5_M, PP3_P, |
| 7 | *LAMP2* | chr21:35821546 | c.123C>A | p.Cys41Ter | NM_002294.2 | Likely pathogenic | Maternal | PVS1_VS, PM2_M |
| 8 | *MYBPC3* | chr11:47361201 | c.2067+1G>A |  | NM_000256.3 | Pathogenic | _ | PVS1_VS, PS4_S, PM2_M |
| 9 | *MYH7* | chr14:23897739 | c.1548C>A | p.Asp516Glu | NM_003280.2 | Likely pathogenic | _ | PS1_S, PM1_M, PM2_M, PP3_P |
| 10 | *MYH7* | chr14:23901007 | c.602T>C | p.Ile201Thr | NM_000257.4 | Pathogenic | _ | PS1_S, PS4_M, PM1_M, PM2_M, PP3_P |
| 11 | *VHL* | chr3:10184560 | c.458_470dup | p.Val158LysfsTer28 | NM_001354723.2 | Likely pathogenic |  | PVS1_VS, PM2_M |
| 12 | *KCNE1* | chr21:35821546 | c.386dup | p.Ter130MetfsTer7 | NM_000219.6 | Likely pathogenic | Paternal | PVS1_VS, PM2_M |
| 13 | *TNNI3* | chr19:55665435 | c.512C>A | p.Ala171Asp | NM_000363.5 | Likely pathogenic | *De novo* | PM2_M, PM5_M, PM6_M, PP3_P |

Identified 13 previously unreported disease-causing variants (4 pathogenic variants and 9 likely pathogenic variants) in 13 patients.

†As presented in the consensus statement of American College of Medical Genetics

‡Parent test was not performed
