# Supplementary material for: Genetic heterogeneity of cardiomyopathy and its correlation with patient care
Source: BMC Med Genomics. 2023 Oct 30;16:270. doi: 10.1186/s12920-023-01639-z (PMC10614404; doi:10.1186/s12920-023-01639-z)
Supplement: Supplementary file 2 — Supplementary Material 2 [file 12920_2023_1639_MOESM2_ESM.docx]

| Supplement table 1. The list of identified variants of uncertain significance in patients with cardiomyopathy | | | | | |  |  |  |
| --- | --- | --- | --- | --- | --- | --- | --- | --- |
| Genes | Phenotype | Position | Nucleotide change | Protein change | Zygosity | GenBank number | Genetic  origin | ACMG |
| *TPM1* | DCM | chr15:63349193 | c.142G>T | p.D48Y | Heterozygosity | NM_001018008.1 | _† | PM2_M, PM5_M, PP3_P |
| *MYH11* | DCM | chr16:15812228 | c.5239G>C | p.E1747Q | Heterozygosity | NM_002474.2 | Paternal | PM2_M, PP3_P |
| *POLG* | DCM | chr15:89876669 | c.317A>T | p.H106L | Heterozygosity | NM_001126131.1 | Maternal | PM2_M, PP3_P |
| *TTN* | DCM | chr2:179436895 | c.46769T>C | p.M15590T | Heterozygosity | NM_003319.4 | Paternal | PM2_M |
| *TNNI3* | DCM | chr19:55668003 | c.118A>G | p.Lys40Glu | Heterozygosity | NM_000363.4 | _ | PM2_M, PP3_P |
| *TNNC1* | DCM | chr3:52486187 | c.137G>A | p.Arg46Lys | Heterozygosity | NM_003280.2 | Paternal | PM2_M |
| *NEXN* | HCM | chr1:78401682 | c.G1426C | p.A476P | Heterozygosity | NM_144573 | _ | PM2_M |
| *TNNC1* | HCM | chr3:52486194 | c.130G>A | p.Val44Met | Heterozygosity | NM_003280.2 | _ | PM2_M, PP3_P |
| *PCCB* | LVNC | chr3:136045705 | c.1151T>G | p.F384C | Heterozygosity | NM_000532.4 | Paternal | PM2_M, PM3_M, PP3_P |
| †Parent test was not performed | | |  |  |  |  |  |  |
