# Supplementary material for: Genetic heterogeneity of cardiomyopathy and its correlation with patient care
Source: BMC Med Genomics. 2023 Oct 30;16:270. doi: 10.1186/s12920-023-01639-z (PMC10614404; doi:10.1186/s12920-023-01639-z)
Supplement: Supplementary file 3 — Supplementary Material 3 [file 12920_2023_1639_MOESM3_ESM.docx]

Supplemental Figure 1. Distribution of cardiomyopathy phenotypes

**
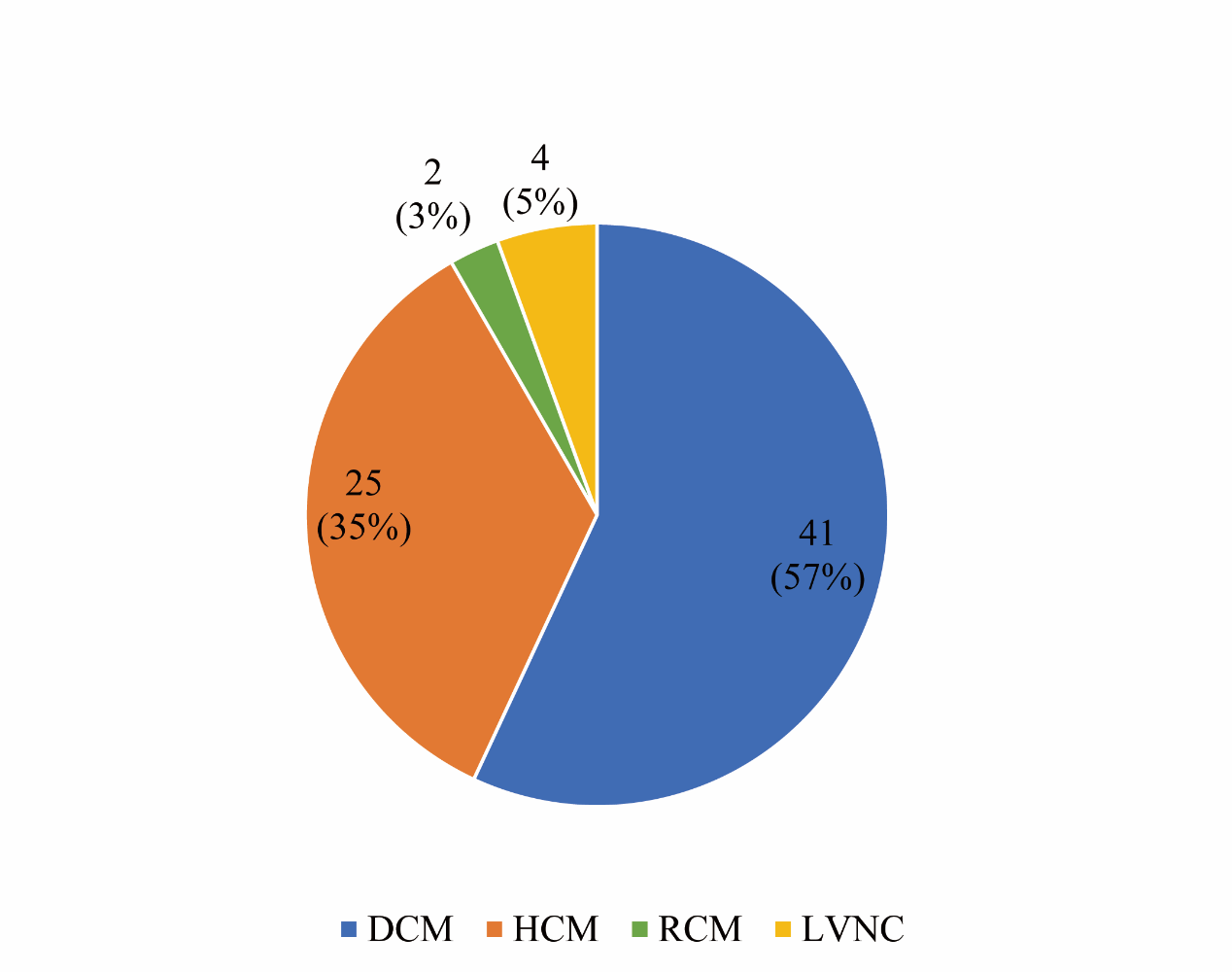
**

DCM (41/72 patients; 56.9%) and HCM (25/72 patients; 34.7%) were the most common subtypes.

Abbreviations: DCM, dilated cardiomyopathy; HCM, hypertrophic cardiomyopathy; RCM, restrictive cardiomyopathy; LVNC, left ventricular non-compaction.
